# Evaluation of Pulse Oximetry Accuracy in a Commercial Smartphone and Smartwatch Device During Human Hypoxia Laboratory Testing

**DOI:** 10.3390/s25051286

**Published:** 2025-02-20

**Authors:** Sara H. Browne, Michael Bernstein, Philip E. Bickler

**Affiliations:** 1 Department of Medicine, University of California San Diego, La Jolla, CA 92093, USA; 2 Specialists in Global Health, Encinitas, CA 92024, USA; 3 Physio Monitor, LLC, San Ramon, CA 94583, USA; 4 Department of Anesthesia, University of California San Francisco, San Francisco, CA 94143, USA; philip.bickler@ucsf.edu

**Keywords:** smartphone, smartphone sensors, smartphone-paired wearables, pulse oximetry, health, human hypoxia testing, FDA clearance

## Abstract

*Background*: The US Food and Drug Administration (FDA) and International Organization for Standardization (ISO) clearance standards for the clinical use of smart device pulse oximetry require in-laboratory human hypoxemia testing in healthy human individuals using arterial blood gas analysis. *Methods*: We evaluated the SpO_2_ measurements of the Samsung smartphone (Galaxy S9/10) and smartwatch (Galaxy 4) at stable arterial oxygen saturations (SaO_2_) between 70 and 100% in 24 healthy participants. Testing followed FDA/ISO-stipulated procedures for pulse oximetry performance validation, which include questionnaire estimation of skin tone based on Fitzpatrick estimation of skin types I–VI. During testing, inspired oxygen, nitrogen, and carbon dioxide partial pressures were monitored and adjusted via partial rebreathing circuits to achieve stable target arterial blood oxygen (SaO_2_) plateaus between 70% and 100%. Arterial blood samples were taken at each plateau, with device SpO_2_ readings taken at each sample extraction. An ABL-90FLEX blood gas analyzer determined arterial blood sample SaO_2_. Bias, calculated from device readings minus corresponding arterial blood measurements, was reported as root mean square deviation (RMSD). *Results*: Combined Participants demographics were: 62.5% female; median age 26 years (range 21–46); and race/ethnicity 16.7% African American, 33.3% Asian, 12.5% multi-ethnic, and 37.5% Caucasian. Fitzpatrick Skin Scale-identified skin tones were: white–fair (I&II), 20.8%; average–light brown (III–IV), 54% and brown–black (V–VI), 25%. There were no adverse events. The RMSD values of SpO_2_ measurements were: smartphone 2.6% (257 data pairs) and smartwatch 1.8% (247 data pairs). *Conclusions*: Device SpO_2_ demonstrated RMSD < 3.0% to SaO_2_, meeting FDA/ISO clearance standards at the time of study. However, additional testing in persons with darker skin tones is necessary. Smartphones and paired wearables, when cleared for clinical use following revision of FDA clearance standards, may expand access to remote pulse oximetry.

## 1. Introduction

Pulse oximetry is a noninvasive method measuring oxygenation status [1]. Current pulse oximeters use red and infrared LED sensors to measure the levels of oxygenated and deoxygenated hemoglobin in the blood to provide an estimate of blood oxygen saturation (SpO_2_) [1]. The gold standard to assess the accuracy of this measurement estimate is the arterial oxygen saturation (SaO_2_) measured by the arterial blood gas (ABG) [2]. Low blood oxygen, termed hypoxemia, depending on its duration, can lead to states of hypoxia, defined as periods where the oxygen available to tissues falls below optimal levels to maintain routine function [3]. While blood oxygen is of critical importance to maintaining homeostatic function, there are multiple variables, including PH and blood flow, that contribute to hypoxia at the tissue level [1]. Consequently, the consensus on what exactly constitutes an abnormal SpO_2_ varies; guidelines from the American Thoracic Society and College of Chest Physicians consider ≤95% at rest or a ≥5% drop with exercise to be abnormal [4].

In clinical practice, pulse oximetry supports the triage and management of symptomatic adults with acute respiratory infections, as well as multiple acute and chronic cardiopulmonary disorders. Pulse oximetry was used during the recent COVID-19 pandemic as an assessment tool to identify patients at risk of poor outcomes and to gauge the severity of infection during Influenza pandemics [5,6,7,8,9,10]. Oxygen saturation (SpO_2_) measurements from patients on home isolation during the recent pandemic were used in community care settings within major healthcare systems to monitor for signs of deterioration, including exercise-induced hypoxia [11,12]. While hypoxia occurs in healthy persons in association with maximal exercise conditions [13,14], COVID-19 infection was associated with hypoxia induced by limited exercise in the absence of radiographic changes [15], and even “silent hypoxia” or minimal shortness of breath in some patients [16]. These reports serve as a reminder that the diagnosis of hypoxemia, even severe hypoxemia, is sometimes missed without objective measures such as non-invasive oximetry or arterial blood gases (ABGs) [17].

The broad clinical utility of SpO_2_ measurement, crucial during respiratory pandemics, highlights the need for accurate pulse oximetry, particularly at home, given the increasing amount of remote patient care [18,19]. The majority of at-home pulse oximeters are inexpensive devices that may be associated with wide variability in accuracy [20]. Pulse oximeters cleared by the FDA as having adequate measurement accuracy for use in clinical settings remain expensive. Global inequity in the distribution of accurate pulse oximetry devices cleared by the FDA or ISO has been well documented by the World Health Organization (WHO), even in hospitalized settings in low- and middle-income countries (LMICs), during the recent pandemic [21,22].

The assessment required by FDA and ISO standards for device SpO_2_ measurement accuracy is based on the evaluation of bias between device SpO_2_ readings and concurrent arterial blood oxygen (SaO_2_) measurements [23,24]. To have clinical utility, oximeters must be able to identify persons with abnormal SpO_2_, particularly those with SpO_2_ < 90%, the most frequent definition of functional hypoxemia [25]. Consequently, FDA/ISO guidance requires testing in healthy individuals during a process of experimentally induced hypoxemia [23,24]. This process is performed in specialist human hypoxia laboratories, where controlled steady-state hypoxemia is induced and arterial blood oxygen saturation between 70% and 100% is targeted following rigorously monitored and controlled procedures standardized in both the FDA 510K clearance and ISO testing requirements [23,24,26,27,28,29]. The design, procedures, and implementation of this testing were made possible by research from expert human hypoxia laboratories, the major contributor being the University of California, San Francisco (UCSF) Human Hypoxia Laboratory (https://hypoxialab.com/about-us/ (accessed on 8 November 2024)) [26,27,28,29], founded by Professor John Severinghaus in 1958 and currently led by the Department of Anesthesia at UCSF. The laboratory conducts research on oxygen transport, oxygen measurement, and high-altitude physiology to understand the effects of hypoxia on humans, and routinely conducts FDA standardized steady-state hypoxemia testing in healthy volunteers [26,27,28,29].

Certain commercial smartphones and paired smartwatches have been manufactured with dedicated hardware using high-grade integrated biosensors with apps that process the sensor data, calculate oxygen saturation and heart rate, and then display the user’s pulse oximetry measurement. Given the increasing need for and global inequity in the availability of pulse oximetry meeting FDA and ISO standards, coupled with the wide availability of smartphones and increasing use of smartwatches, we performed detailed human hypoxia testing using arterial blood samples to determine the accuracy of integrated pulse oximetry in a commercial smartphone (Samsung Galaxy S9/10) and a commercial smartwatch (Samsung Galaxy Watch 4). These devices are in wide global circulation: Samsung Galaxy S9/10 smartphones had an estimated 116 million devices circulating in 2020, and the Samsung Galaxy Watch 4 had an estimated 3 million devices in circulation in 2022 [30,31]. We performed an experimental vital sign measurement accuracy validation study in the human hypoxia laboratory following strict adherence to FDA procedural requirements for pulse oximetry clearance for use in clinical medical settings. We hypothesized that the devices would either approach or meet the standards specified at the time of testing.

## 2. Materials and Methods

Study Design: Prospective in vivo invasive laboratory procedures were performed on healthy volunteers to validate the SpO_2_ accuracy specifications of the pulse oximeter system by comparing each value from the device and a simultaneous value from co-oximetry of an arterial blood sample. This study type compares oximetry measurements taken by a device to the recognized “gold standard” measurement, which is the arterial blood gas oximetry. Studies were performed in the University of California, San Francisco Human Hypoxia Laboratory following the FDA procedures and data reporting requirements in place at the time of study. FDA requirements specified 10 or more healthy subjects varying in age and gender with a range of skin pigmentations (including at least 2 darkly pigmented participants or 15% of the participant pool, whichever was larger) [23,24]. The standards for reporting measurement accuracy guidelines included reports of the root mean square deviation (RMSD) of measurements taken simultaneously by the device and the arterial blood gas in a range of SaO_2_ 70–100% based on 200 or more paired observations (pulse oximeter, co-oximeter) with Bland Altman error plots, including mean bias with upper 95% and lower 95% limits of agreement [23,24]. These FDA standards are undergoing review. As this study did not assign subjects to different treatments for the assessment of health outcomes, it does not meet the definition of a clinical trial that requires registration at clinicaltrials.gov.

### 2.1. Test Device Descriptions

**Figure 1:**
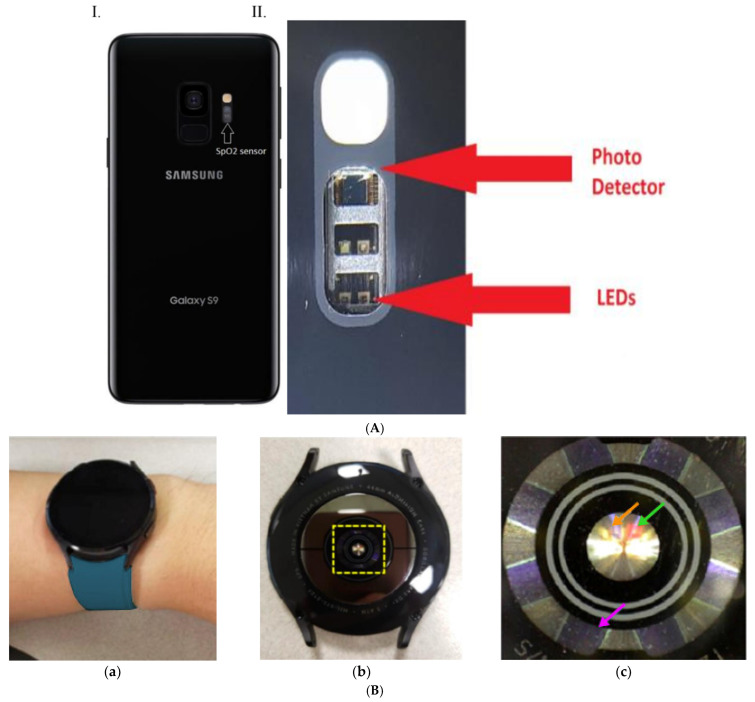
(**A**): (I) The location of the SpO_2_ sensor on the back of the smartphone, making it clear that the sensor is entirely separate from the other phone devices such as the camera. In the expanded view (II), the dedicated photo sensor and LEDs for the pulse oximetry function are shown. The device has a total of 4 LEDs, 2 of which are reserved for future additional functionality. The arrow indicates the 660 nm red and 910 nm infrared LEDs used in this measurement. The photodetector is connected to an extremely low-noise analog channel, allowing for measurement on a broad range of skin colors. (**B**): The Galaxy Watch 4 (GW4) used in this study. (**a**): Ideal GW4 placement on the wrist, avoiding the ulnar styloid process of the participants. (**b**): View of the side of the watch in contact with the skin, showing placement of the light-emitting diodes and photodetector (LED-PD) module (contained in the yellow rectangular area). (**c**): Magnified view of the LED-PD module with arrows pointing to LEDs and PDs. Photodiodes are located radially, with the purple arrow indicating one of the eight PDs. Infrared LEDs are pointed out by the orange arrow and red LEDs by the green arrow [33].

*Smartphone:* A Samsung S9+ smartphone (Samsung Electronics, Suwon-si, Republic of Korea) containing Maxim Integrated biosensors, part number MAX86916 (Maxim Integrated, San Jose, CA, USA), associated with the proprietary Samsung Health App (https://www.samsung.com/us/apps/samsung-health/, accessed on 18 September 2020), was the smartphone device tested. The test instrument has phone biosensors which include a separate specialized dedicated photodetector with 2 precision wavelengths of 660 nm red and 910 nm infrared LEDs used to take measurements, which are connected to an extremely low-noise analog channel dedicated to the oximetry function (See Figure 1A). The arrangement of and distance between S9 pulse oximeter LEDs and the photodiode in the optical sensor module are shown in Supplementary Figure S1A, and a full technical description of the integrated optical sensor module is available (see reference [32]). The associated app processes the sensor data, calculates saturation and heart rate, and then displays the measurement for viewing and recording. To enable test participants to hold their fingers continuously in the same position over a 30–40-min period of testing, a silicone boot attached to a plastic cell phone case was used during testing (see Supplementary Figure S1B). Use of the boot was solely to support finger position over the extended time period of testing. There was no requirement to reduce ambient light with this finger pulse oximeter; finger placement for readings took approximately 30 s, and no readings were displayed unless the PPG quality parameters were met.

*Smartwatch:* A Samsung Galaxy smartwatch (Samsung Electronics, Suwon-si, Republic of Korea) was the wrist device tested. The watch device, shown in Figure 1B, is equipped with a reflectance pulse oximeter module composed of light-emitting diodes and photodetectors that capture photoplethysmography (PPG) [33]. GW4 captures the PPG signal with a sampling frequency of 25 Hz for each wavelength, and SpO_2_ is calculated every second with output data displayed as integers [33,34,35]. The similarity of repetitive pulse waveforms is continuously monitored to filter out inconclusive conditions, and if the PPG signal quality is considered low, the output value is not reported [33,34,35]. A well-defined alternating component (AC)/direct component (DC) method is used to calculate SpO_2_. Briefly, noise in the PPG waveform is eliminated using a low-pass filter (fc = 12.5 Hz). Then, the AC and DC are measured based on the recent PPG waveform within a window. The perfusion index (PI) and R-value are calculated as PI_λ_ = AC_λ_/DC_λ_ and R-value = PI_λRed_/PI_λIR_, respectively. The SpO_2_ level is estimated based on the R-value using the predefined calibration information [33].

During human hypoxia testing, intermittent or “spot check” SpO_2_ measurement accuracy was evaluated, in which case both devices performed pulse oximetry calculations when triggered.

### 2.2. Procedures

Evaluation of test device oximeter performance was performed using controlled steady-state hypoxemia at the UCSF Human Hypoxia Laboratory. Detailed descriptions of the procedures followed to test pulse oximetry device accuracy following FDA/ISO guidance are also available in prior publications from our laboratory and a collaborating commercial laboratory [23,24,26,27,28,29].

Each device was tested individually in a separate set of investigations. The work was sanctioned by the UCSF Human Research Protection Program (https://irb.ucsf.edu/human-research-protection-program-hrpp (accessed on 8 November 2024)), with protocol approval #10-00437 (Smartphone), and approval # 21-35637 (smartwatch), conforming to all internationally accepted standards for the protection of human subjects. Participants were recruited from a database of healthy volunteers held by the UCSF Human Hypoxia Laboratory based on their availability, at convenient testing times. Participants were required to have normal hemoglobin levels (hemoglobin ≥ 10 gm/dL) and to be physically active, healthy, non-smoking individuals of ages 22–46. Exclusion criteria included, but were not limited to, age ≥ 50 or age < 21, systemic illness, and obesity. Written informed consent was obtained. During the informed consent process, participants were informed of the potential risks of having radial artery cannulation with continuous use of up to 40 min and the potential risks of respiring air with reduced oxygen tension. All controlled human protocols were conducted by a Board-Certified Anesthesiologist, who continuously monitored and asked participants to indicate any discomfort and their comfort level. If discomfort was indicated, the procedure was terminated.

Volunteers who met the inclusion criteria and completed the written informed consent processes were compensated USD 200.00 for their participation in this steady-state hypoxia study. All data collected during the study were de-identified and stored in a protected computerized database following Good Clinical Practice and under the approval of the University of California, San Francisco (UCSF) Human Research Protection Program.

Demographic data were self-reported by participants, and height and weight were measured in the laboratory by research personnel. An estimate of the Fitzpatrick Skin Scale type was obtained based on the participants’ completion of a questionnaire scale following the requirements of the FDA and ISO [23,24,36,37]. Participants were in a 30°, head-up, semi-recumbent position for the study. After local anesthesia with lidocaine, a 22-gauge radial arterial cannula was placed in either the left or right wrist of each participant for arterial blood sampling and blood pressure monitoring. A Masimo Radical 7 (Masimo Inc., Irvine, CA, USA) with a DCI clip-type adult reusable finger sensor or ear clip sensor, as well as Nellcor N-595 (Medtronic Inc., Minneapolis, MN, USA) with a reusable finger clip sensor (Nellcor DS-100A), were placed on one of the middle 3 fingers or the ear. Probes were repositioned as necessary to ensure proper placement throughout the study. No subjects wore nail polish. Data from the pulse oximeters were recorded at 2 Hz from the instruments’ serial output ports using a computer running LabVIEW 15.0 (National Instruments, Austin, TX, USA). Data from the Masimo were transmitted at 1 Hz, while the data from the Nellcor (Medtronic, MN, USA) were transmitted at 0.5 Hz. Recorded data included SpO_2_, heart rate, and perfusion index from each device. Blood gas analysis to determine oxyhemoglobin saturation (SaO_2_) was performed using ABL-90 multi-wavelength oximeters (Hemoximeter, Radiometer, Copenhagen, Denmark). These instruments contained factory-certified calibration standards and quality control algorithms.

Each participant had two control blood samples taken at the beginning of each experiment while breathing the room air. Hands/arms with pulse oximeters were maintained motionless on arm boards. For smartphone testing, to enable the test participants to hold their fingers continuously in the same position over a 30–40-min period of testing time, a silicone boot attached to a plastic cell phone case was used. For smartwatch testing, the device was placed on the wrist, avoiding the ulnar styloid process and tightened against the skin, with the strap adjusted by an experienced laboratory technician to obtain a high-quality PPG signal [33,34]. The technician ensured persistent strap tightness to provide a high-quality PPG signal throughout testing (See [33,34]).

Hypoxemia was then induced to different and stable levels of oxyhemoglobin saturation (between 70% and 100%) by the study anesthesiologist, who adjusted participants’ inspired gas mixtures using controlled mixtures of nitrogen, air, and carbon dioxide obtained by adjusting gas flows with valves according to breath-by-breath estimates of oxygen saturation calculated from end-tidal PO_2_ and PCO_2_, which were displayed on a screen using LabVIEW 2015. Stable SaO_2_ plateaus between 70% and 100% were targeted by the study physician who adjusted the inspired gas mixture. Each plateau level of oxyhemoglobin saturation was maintained for at least 30 s or until reference pulse oximeter readings were stable. Two arterial blood samples were then obtained approximately 30 s apart. Each stable plateau, therefore, was maintained for at least 60 s with SpO_2_ values of 100% initially reduced to 70% over 10 plateaus per participant. Samples were obtained at the saturation plateaus across this span. The SpO_2_ readings for the two devices were triggered at the time of each sample, and the SpO_2_ was held until each device completed the reading. All data were stored securely and provided for analysis. Reference data were measured and recorded independent of the study physicians. Only samples with stable SpO_2_ readings (i.e., fluctuation < 2% per minute) were included in the analysis. No subjects had methemoglobin (MetHb) or carboxyhemoglobin (COHb) outside the normal range of 0–3% or 1–3%, respectively, as measured by the hemoximeter (Radiometer ABL90 Flex). At least 200 data points were collected for each type of oximeter and probe combination studied.

### 2.3. Statistical Analysis

Pulse oximeter data were taken as one reading corresponding to the point of arterial blood analysis. Individual data points may have been missed or excluded for dropped signals or failure of the oximeter signal to achieve an appropriate plateau. Bias was computed as test device readings minus the corresponding arterial blood sample values. Bias was reported as the root mean square deviation (RMSD). Plots following Bland and Altman with adjustments for multiple measurements for each participant according to the “Method Where the True Value Varies” were generated [38]. The FDA guidance prescribes the use of a Bland Altman error plot with specifically calculated limits of agreement [23,24,38]. Data were plotted as hemoximeter data (SaO_2_) vs. pulse oximeter bias (SpO_2_–SaO_2_). A different marker was used for each study subject. Linear regression was calculated for all participants combined for each device tested, and the equation with R^2^ was included on a Bland Altman plot.

## 3. Results

### 3.1. Demographics

*Smartphone:* Twelve participants completed the study and had the following demographics: 66.7% female; median age 26 years (range 21–31); race/ethnicity 25% African American, 33% Asian, 8.3% multiethnic, and 33.3% Caucasian; and Fitzpatrick scale type I 8.3%, type II 16.7%, type III 33.3%, type IV 16.7%, type V 8.3%, and type VI 16.7%. Demographics for each individual participant are shown in Table 1A. There were no adverse events.

*Smartwatch:* Twelve participants completed the study and had the following demographics: 58.3% female; median age 25.5 years (range 22–46); race/ethnicity 8.3% African American, 33.3% Asian, multiethnic 16.7%, and Caucasian 41.7%; and Fitzpatrick scale type II 16.7%, type III 16.7%, type IV 41.7%, type V 16.7% and type VI 8.3%. Demographics for each individual participant are shown in Table 1B. There were no adverse events.

### 3.2. Test Device Bias

**Figure 2:**
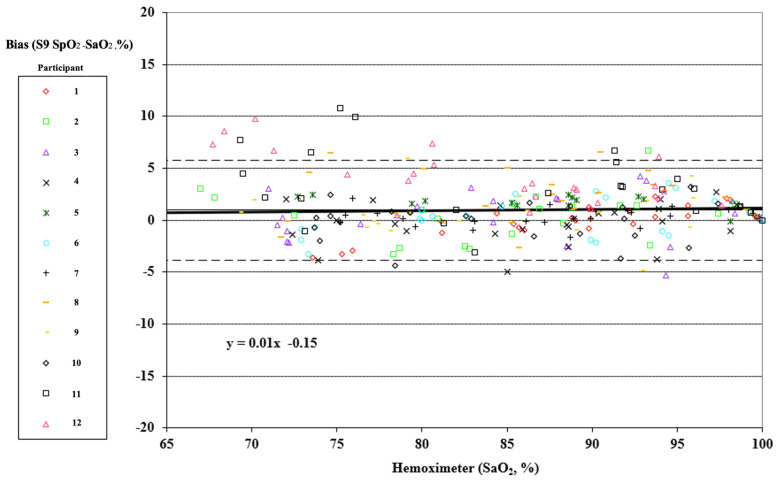
Plot of data collected from 12 human volunteers during tests of the smartphone SpO_2_ measurement bias from arterial blood oxygen saturation (SaO_2_) measurements. Each point on the plot represents paired blood oxygen levels recorded by the hemoximeter associated with the smartphone reading. The dashed lines show the upper and lower limits of agreement as per Bland Altman 2007 [33].

*Smartphone:* During testing, no plateaus were rejected for lack of stability between the first blood sample and the second. Eighteen readings from the device under testing were rejected because the results were delayed by more than 15 s past the sample time. The RMSD of the 257 remaining data points based on blood sample analysis obtained from 12 participants tested was 2.6%. Figure 2 displays a modified Bland Altman plot of the entire data set. Data are plotted as hemoximeter data (SaO_2_) versus pulse oximeter bias (SpO_2_–SaO_2_), with a different marker used for each participant. Linear regression is included for all participants combined, and the equation with R^2^ is shown on the plot. Mean bias is reported as a solid horizontal line, and the upper and lower limits of agreement (mean bias ± 1.96·SD) are shown by dashed horizontal lines.

**Figure 3:**
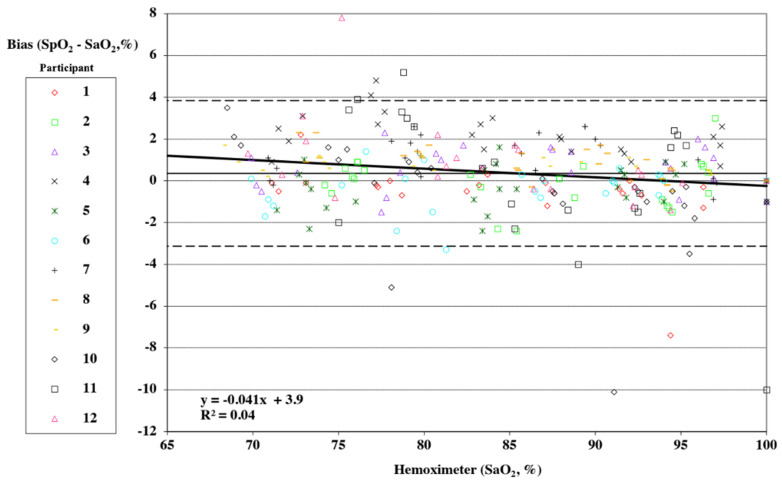
Plot of data collected from 12 human volunteers during testing of the smartwatch SpO_2_ measurement bias from arterial blood oxygen saturation (SaO_2_) measurements. Each point on the plot represents paired blood oxygen levels recorded by the hemoximeter associated with the smartwatch reading. The upper and lower limits of agreement as per Bland Altman 2007 [33] are shown by the dashed lines.

*Smartwatch*: In this study, no plateaus were rejected for lack of stability between the first sample and the second. Twenty-eight readings from the device under testing were rejected because the results were delayed by more than 15 s past the sample time. The RMSD of over 247 data points based on blood sample analysis obtained from the 12 participants tested was 1.8%. Figure 3 displays a modified Bland Altman plot of the entire data set. Data are plotted as hemoximeter data (SaO_2_) versus pulse oximeter bias (SpO_2_–SaO_2_), with a different marker used for each participant. Linear regression is included for all participants combined, and the equation with R^2^ is shown on the plot. Mean bias is reported as a solid horizontal line, and the upper and lower limits of agreement (mean bias ± 1.96·SD) are shown by dashed horizontal lines.

### 3.3. Discussion

*Interpretation of findings*: Evaluation of oximeter performance during controlled steady-state hypoxemia revealed that the smartphone readings had an RMSD error of 2.6% from over 257 simultaneous ABGs. The RMSD of <3.0% RMSD of arterial SpO_2_ values met the FDA/ISO accuracy requirements for clinical pulse oximetry at the time these studies were conducted [23,24,38]. Evaluation of oximeter performance during controlled steady-state hypoxia revealed that smartwatch readings had an RMSD error of 1.8% from 247 simultaneous ABGs. These findings indicate that existing embedded dedicated hardware and preloaded apps in these devices can take accurate clinical-grade pulse oximeter readings. The implications of our findings regarding global access to accurate pulse oximetry are considerable.

This is the first report of smartphone-derived pulse oximetry measurements that have met the FDA/ISO accuracy requirements applicable at the time of study using arterial blood samples. Up to this point, studies evaluating smartphone pulse oximetry have shown highly variable accuracy, with the minority supporting any clinical use. Because of this, we will discuss prior smartphone studies in some detail. None of the prior smartphone studies performed human laboratory testing using ABGs as the oxygen saturation reference during FDA/ISO-stipulated hypoxemia protocols. In addition, prior smartphone studies were conducted using different protocols within different patient populations, utilizing devices containing widely variable hardware. Jordan et al. compared pulse oximetry apps downloaded onto iPhones to reference monitors within an emergency room setting and found that the apps provided inaccurate measurements, with a sensitivity for detection of SpO_2_ < 94% on the reference monitor ranging from 0 to 69% [39]. Two of these apps utilized the onboard light and camera lens (Pox and Ox), and one used an external device that plugged into the iPhone. Alexander et al. evaluated two other smartphone apps (Pulse Oximeter and Pulse Oximeter Pro) downloaded onto an iPhone using an onboard light and a camera lens to perform SpO_2_ measurements, predominantly in a pre-operative setting [40]. A comparison of these apps with clinical devices utilized by the Dept. of Anesthesia showed similar mean and median values, but wide and highly significant variance in measurements [35]. Tayfur et al. compared SpO_2_ measurements from 101 hospitalized patients (43% having pulmonary disease) obtained from Samsung Galaxy S8 smartphones and simultaneous ABGs. They reported the measurements to be highly correlated and to have low bias with narrow levels of agreement [41]. Modi et al. conducted 6-min walk pre- and post- oximetry testing to perform a comparison of a Masimo-radical7 device, a clip Kenek sensor connected to an iPhone, and a Samsung Galaxy 8 [42]. They reported a correlation between SpO_2_ measurements (r = 0.62–0.72, *p* < 0.001) and provided values from 28 out of 47 participants using a Bland Altman analysis that used an average of the SpO_2_ obtained by the FDA-approved Masimo-radical7 reference device and the smartphone readings as the “true” SpO_2_, making these data difficult to evaluate. Browne et al. performed clinical studies that utilized a repeated-measure, nested factorial design to evaluate the accuracy and precision of a smartphone model containing high-grade biosensors in comparison to Welch Allyn reference units on 320 participants and found the accuracy and precision to be equivalent to hospital-grade clip sensors [43]. This smartphone was also tested in the Human Hypoxia Laboratory and found to have an RMSD of 2.02% versus a portable FDA/ISO-approved reference device, but no information was available against the gold-standard ABGs [43].

The major determinant of accuracy differences observed across these smartphone studies reflects differences in the quality of the underlying biosensor hardware, proprietary algorithms, and device design within the smartphones tested. Pulse oximeters used in hospital settings rely on both the reflection and transmission of light through cutaneous tissues such as the finger or ear lobe. Smartphone systems utilize reflected light detected by a sensor on the same surface as the emitter [44]. How the reflected light signal is obtained and analyzed is critical. Poorly performing pulse oximetry apps use the onboard light and camera lens to obtain reflected light to detect PPG signals and conformational changes associated with hemoglobin binding. In the smartphones tested, it is likely that the camera-associated sensors have relatively high signal-to-noise ratios and may even block near-infrared light [44]. In contrast, the smartphone evaluated in our study had dedicated hardware specifically for pulse oximetry (see Figure 1A), with placement entirely separate from other in-phone devices such as the camera. The smartphone pulse oximeter tested displayed readings within 30 s of finger placement and did not report values unless signal quality thresholds were met. The measurement accuracy and precision of finger placement pulse oximetry using a smartphone that contains the same high-grade biosensor suite as the S9/S10 Galaxy series was evaluated using 1140 readings performed simultaneously with in-hospital (Welch Allyn) instruments. In a clinical patient population of wide age range and reasonable racial/ethnic diversity, accuracy and precision were equivalent to hospital devices [43]. Studies testing similar separate high-grade biosensor hardware have reported high smartphone SpO_2_ measurement accuracy [41,43]. This detailed discussion indicates that it is vital for studies performed in clinical settings to understand and specify the type of embedded biosensor hardware as well as the site of placement within remote devices when assessing oximetry performance, not simply the make and model of the smartphone device.

In contrast to studies using smartphones alone, prior publications have reported wrist-based devices that pair with smartphone apps, with pulse oximetry meeting *current* FDA/ISO accuracy clearance thresholds. One watch device reports a performance specification for SpO_2_ measurement accuracy of 2% [45]. An alternative device, with testing data available for review obtained from Human Hypoxia Laboratory testing in comparison to concurrent ABG measurements, demonstrated an RMSE of 2.97% [34]. Determinants of PPG quality at the wrist are being clarified [46]. Recent reports of the performance of wrist-worn reflectance pulse oximetry during sleep have also indicated potential utility for obstructive sleep apnea (OSA) evaluation based on the accuracy of wrist-based pulse oximetry [33,47,48]. One of these studies included a bias analysis of overnight continuous SpO_2_ measurements obtained by the same commercial smartwatch which we tested in comparison to hospital-grade polysomnography (Nihon Kohden) pulse oximetry and reported an RSME of <3.0% [48]. In addition, the use of artificial intelligence-enhanced sensing has been shown to improve performance in wrist-worn wearables [49].

### 3.4. Implications for Clinical Assessment

The rapid surge in demand for pulse oximetry during the recent COVID-19 pandemic was captured by market analysis and media reports indicating that consumer purchases of home pulse oximeters increased by 500% within the first 8 months [50]. Given the reality of recurrent respiratory pandemics and the increasing use of remote care to address the management of acute infection and chronic cardiopulmonary disease, increased demand for at home pulse oximetry will likely persist. The foremost concern of experts regarding home pulse oximetry is the accuracy of SpO_2_ measurements, particularly as saturation falls below 90% [20,44]. The cost of FDA/ISO-cleared pulse oximeters remains high, and within LMIC environments, actual medical clinics and even hospital settings have been documented as having limited or no access to pulse oximetry [21,22]. As data on the accuracy of inexpensive stand-alone oximeters are limited, the Open Oximetry Project (https://openoximetry.org/) led by the UCSF Human Hypoxia Laboratory was launched in 2021 to evaluate device accuracy during hypoxemia. The data we present, obtained independently from this project, now provide unequivocal evidence that it is possible for commercial smartphone and paired smartwatch devices, with appropriate dedicated hardware such as the devices tested, to reach *current* FDA/ISO accuracy requirements for clinical use. This means that multiple millions of accurate pulse oximeters may be made available to consumers purchasing such smart devices with the potential to improve and expand at-home data collection and monitoring in both acute and chronic cardiopulmonary disorders. In terms of user data, there is evidence that a smartphone model could be used by a wide range of adult persons to perform repeated pulse oximeter spot checks with accuracy and precision equivalent to hospital-grade clip sensors [43]. Evidence that users can utilize smartwatches to obtain spot checks with accuracy and precision equivalent to hospital-grade clip sensors is currently lacking. Pulmonologists’ practical guidance for patients using at-home oximetry monitoring recommends that devices should provide indications of pulse signal strength and that measurements should be taken and recorded at rest two to three times a day [44]. The devices tested do not report oximetry unless the pulse signal strength is adequate, and thus meet this guidance. Use of an associated smartphone app is necessary to store and potentially share oximetry reading data.

### 3.5. Study Limitations

The most important caveat to our study findings is that FDA/ISO guidance is undergoing re-evaluation based on reports during the COVID-19 pandemic that the accuracy of pulse oximetry may vary based on race/ethnicity related to skin pigmentation [51,52,53,54,55]. While the human hypoxia studies we conducted followed FDA guidelines at the time of the study’s performance, the variance in individual participant readings obtained during device testing indicates that further evaluation is needed to understand performance accuracy in persons with darker skin tone. The Open Oximetry Project has recently reported extensive data indicating that the current standards do not adequately account for the impact of skin pigmentation on device performance and that visual skin pigmentation scales, such as the currently required Fitzpatrick scale, should be replaced [50,51].

This study, in accordance with FDA/ISO specifications, used healthy volunteers under steady-state hypoxia testing (SpO_2_ approximately 70–100%), and thus provides no data with accuracy below 70%. Data from healthy volunteers provide no information on the accuracy of SpO_2_ measurement under the conditions of reduced extremity pulsatile blood flow associated with vasoconstriction due to Raynaud’s, hypotension, or peripheral vascular disease. The latter is common in persons with diabetes and coronary heart disease. Furthermore, measurement accuracy in the presence of diseases associated with hemoglobinopathies, such as sickle cell or thalassemia, or where elevated carboxyhemoglobin (potentially present in heavy smokers) or methemoglobin (associated with the use of chloroquine and sulfonamides) may occur, is unknown. This study does not provide any evidence on the use of this system in children. Finally, the tested system provided SpO_2_ spot checks only; our study did not evaluate continuous pulse oximetry.

## 4. Conclusions

Our findings indicate that the commercial smartphone and smartwatch devices with the appropriate dedicated hardware tested under conditions of induced hypoxemia were sufficiently accurate to reach FDA/ISO clearance for use in clinical settings at the time of the study. However, the current standards are undergoing re-evaluation based on evidence that insufficient data are generated to adequately capture reliability in persons with darker skin tones [54,55]. Thus, while our accuracy findings are promising, additional testing is necessary to adequately assess whether clinical utility is demonstrated in persons with darker skin tones. As data accumulate indicating that commercial smart devices with appropriate dedicated hardware meet revised clinical regulatory oximetry standards, multiple millions of accurate pulse oximeters will be available to consumers purchasing these devices, with the potential to improve and expand at-home data collection and monitoring in both acute and chronic pulmonary disorders.

## Figures and Tables

**Table 1:** Demographics of individual participants during human testing of pulse oximetry accuracy within the smartphone and smartwatch models tested.

Participants	Gender	Age	Height (cm)	Weight (kg)	Race/Ethnicity	Fitzpatrick Scale
A: Smartphone						
1	Female	26	160.0	52.3	Asian	Type III
2	Male	24	180.3	71.8	African American	Type VI
3	Female	21	175.3	68.2	African American	Type VI
4	Female	27	154.9	54.5	Asian	Type III
5	Female	26	177.8	77.3	Caucasian	Type II
6	Female	29	167.6	67.3	African American	Type V
7	Male	31	198.1	95.5	Caucasian	Type IV
8	Female	28	175.3	57.7	Caucasian	Type II
9	Male	26	167.6	62.7	Asian	Type III
10	Female	22	157.5	56.4	Multi-ethnic	Type III
11	Female	27	162.6	45.5	Caucasian	Type I
12	Male	24	175.3	63.6	Asian	Type IV
B: Smartwatch						
1	Female	22	165.0	52.0	Asian	Type IV
2	Male	24	160.0	63.6	Asian	Type IV
3	Female	30	172.7	55.5	Caucasian	Type III
4	Female	28	165.1	59.1	Caucasian	Type II
5	Male	22	180.0	68.0	Caucasian	Type IV
6	Female	22	157.5	48.2	Asian	Type V
7	Female	23	165.1	59.1	Asian	Type V
8	Male	26	177.0	77.0	Caucasian	Type II
9	Female	26	160.0	59.1	Multi-ethnic	Type IV
10	Male	28	175.3	68.2	Multi-ethnic	Type IV
11	Male	46	180.3	94.5	African American	Type VI
12	Female	25	163.0	72.0	Caucasian	Type III

## Data Availability

Data generated during the study supporting reported results can be obtained by application to https://hypoxialab.com/contact/ (accessed on 1 November 2024), attention M. Bernstein.

## Supplementary Materials

The following supporting information can be downloaded at: https://www.mdpi.com/article/10.3390/s25051286/s1, Figure S1: A: Provides a schematic of the pulse oximeter biosensors within the S9 (Samsung Electronics, Suwon-si, Republic of Korea) with distances for the LEDs and Photodiode detector (part number MAX86916 Maxim Integrated/Analogue Devices Inc., San Jose, CA, USA) associated with the proprietary Samsung Health App. B: Shows the silicone boot attached to a plastic cell phone case that was utilized in this test to hold the finger consistently in place during 30–35 min of Laboratory testing.
