# Supplementary material for: Evaluation of Pulse Oximetry Accuracy in a Commercial Smartphone and Smartwatch Device During Human Hypoxia Laboratory Testing
Source: Sensors (Basel). 2025 Feb 20;25(5):1286. doi: 10.3390/s25051286 (PMC11902706; doi:10.3390/s25051286)
Supplement: Supplementary file 1 [file sensors-25-01286-s001.zip › sensors-3333529-supplementary.pdf]

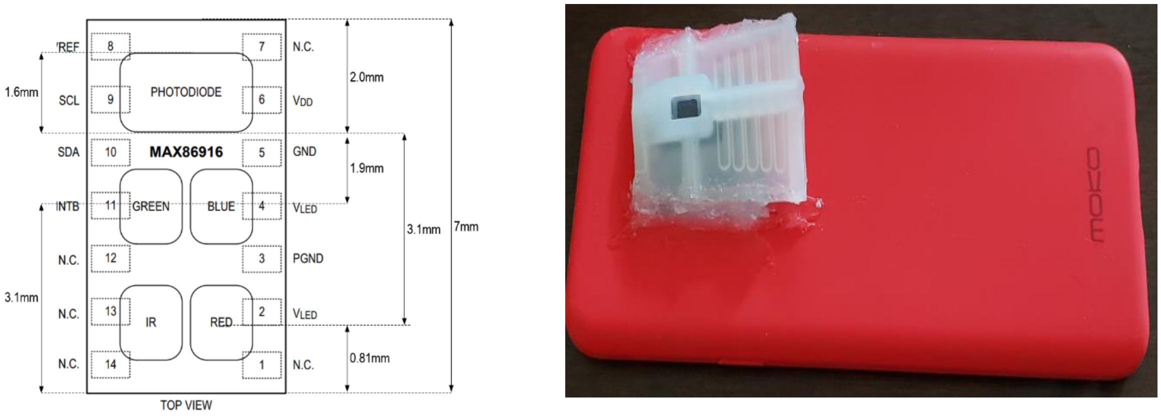

A B

Supplementary Figure S1. A: Provides a schematic of the pulse oximeter biosensors within the S9 (Samsung Electronics, Suwon-si, Korea) with distances for the LEDs and Photodiode detector (part number MAX86916 Maxim Integrated/ Analogue Devices Inc. San Jose, CA) associated with the proprietary Samsung Health App. B: Shows the silicone boot attached to a plastic cell phone case that was utilized in this test to hold the finger consistently in place during 30-35 mins of Laboratory testing.
